# 7^th^ Brazilian Guideline of Arterial Hypertension: Chapter 13 - Resistant Arterial Hypertension

**DOI:** 10.5935/abc.20160163

**Published:** 2016-09

**Authors:** MVB Malachias, CIS Rodrigues, E Muxfeldt, GF Salles, H Moreno Júnior, M Gus

## Definition and epidemiology

Resistant AH (RAH) is defined as uncontrolled office BP despite the use of at least three antihypertensive drugs at appropriate doses, including preferably one DIU, or as controlled BP using at least four drugs.^1-3^ Because it does not include the systematic assessment of therapy and adherence, that situation is better defined as apparent RAH (pseudoresistance). Identification of true RAH is fundamental to establish specific approaches.^2^ Population-based studies have estimated a 12% prevalence in the hypertensive population.^2^ In Brazil, the ReHOT study assesses prevalence and therapeutic choice.^4^ Refractory hypertension is defined as uncontrolled BP using at least five antihypertensive drugs,^5^ and corresponds to 3.6% of resistant hypertensive individuals. To diagnose RAH, ABPM is required, as well as systematic assessment of adherence. (GR: I; LE: C).

## Associated factors

Causative factors are as follows: higher salt sensitivity, increased blood volume (higher sodium intake, CKD or inappropriate diuretic therapy), exogenous substances that raise BP, and secondary causes (OSAHS, primary aldosteronism, CKD, and renal artery stenosis).^1,3,6^ The characteristics of RAH are: more advanced age, African ancestry, obesity, MS, DM, sedentary lifestyle, chronic nephropathy, and LVH.^1,3^

The pathophysiological aspects related to resistance are as follows: (i) sympathetic and RAAS hyperactivity; (ii) vascular smooth muscle proliferation; (iii) sodium retention; and (iv) activation of proinflammatory factors.^1,7^ Greater endothelial dysfunction and arterial stiffness are present.^8^ In ABPM, there is high prevalence (30%) of WCE and attenuation of nocturnal BP dipping.^9^ The prevalence of black ethnicity, DM and albuminuria is higher among refractory hypertensive individuals.^5^

## Diagnostic investigation

### Pseudoresistance

Pseudoresistance is due to poor BP measurement technique, low adherence to treatment and inappropriate therapeutic regimen.^1,2,10^ Studies have shown that 50-80% of the patients fail to adhere to treatment completely or partially.^10-12^ The diagnosis of RAH should only be established after inclusion of an appropriate DIU^13^ and adjustment of the antihypertensive regimen.^12^

### Complementary tests

Blood biochemistry, urinalysis and ECG should be requested at the time of diagnosis, and repeated at least once a year.^1,12^ Echocardiogram and retinal exam, when available, should be repeated every 2 to 3 years.

### Secondary causes

Secondary causes are common in RAH,^6^ OSAHS being the most prevalent (80%, and 50% with moderate-severe apnea),^14^ followed by hyperaldosteronism (20%, mainly adrenal hyperplasia)^15^ and renal artery stenosis (2.5%).^6^ Other secondary causes should only be investigated in the presence of suggestive clinical findings.^6^

### ABPM and HBPM

Although the diagnosis of RAH is based on office BP measurement,^1^ BP assessment by using ABPM or HBPM is mandatory for the initial diagnosis and clinical follow-up.^1,9,16,17^ It is estimated that 30-50% of resistant hypertensive individuals have normal outside-the-office BP levels.^9,12,16^ The diagnosis obtained on ABPM defines diagnostic and therapeutic management (Chart 1).^1,12,16^

**Chart 1:** Major causes of secondary AH, signs and diagnostic screening

Clinical findings	Diagnostic suspicion	Additional studies
Snoring, daytime sleepiness, MS	OSAHS	Berlin questionnaire, polysomnography or home respiratory polygraphy with at least 5 episodes of apnea and/or hypopnea per sleep hour
RAH and/or hypopotassemia (not necessary) and/or adrenal nodule	Primary hyperaldosteronism (adrenal hyperplasia or adenoma)	Measurements of Aldo (>15 ng/dL) and plasma renin activity/concentration; Aldo/renin > 30. Confirmatory tests (furosemide and captopril). Imaging tests: thinsliced CT or MRI
Edema, anorexia, fatigue, high creatinine and urea, urine sediment changes	Parenchymal kidney disease	Urinalysis, GFR calculation, renal US, search for albuminuria/proteinuria
Abdominal murmur, sudden APE, renal function changes due to drugs that block the RAAS	Renovascular disease	Renal Doppler US and/or renogram, angiography via MRI or CT, renal arteriography
Absent or decreased femoral pulses, decreased BP in the lower limbs, chest X ray changes	Coarctation of the aorta	Echocardiogram and/or chest angiography via CT
Weight gain, decreased libido, fatigue, hirsutism, amenorrhea, moon face, “buffalo hump”, purple striae, central obesity, hypopotassemia	Cushing’s syndrome (hyperplasia, adenoma and excessive production of ACTH)	Salivary cortisol, 24-h urine free cortisol and suppression test: morning cortisol (8h) and 8 hours after administration of dexamethasone (1 mg) at 24h. MRI
Paroxysmal AH with headache, sweating and palpitations	Pheochromocytoma	Free plasma metanephrines, plasma catecholamines and urine metanephrines. CT and MRI
Fatigue, weight gain, hair loss, DAH, muscle weakness	Hypothyroidism	TSH and free T4
Increased sensitivity to heat, weight loss, palpitations, exophthalmos, hyperthermia, hyperreflexia, tremors, tachycardia	Hyperthyroidism	TSH and free T4
Renal lithiasis, osteoporosis, depression, lethargy, muscle weakness or spasms, thirst, polyuria	Hyperparathyroidism (hyperplasia or adenoma)	Plasma calcium and PTH
Headache, fatigue, visual disorders, enlarged hands, feet and tongue	Acromegaly	Baseline IGF-1 and GH and during oral glucose tolerance test

OSAHS: obstructive sleep apnea-hypopnea syndrome; Aldo: aldosterone; RAH: resistant arterial hypertension; GFR: glomerular filtration ratio; APE: acute pulmonary edema; RAAS: renin-angiotensin-aldosterone system; CT: computed tomography; ACTH: adrenocorticotropin; TSH: thyroid stimulating hormone; PTH: parathormone; IGF-1: insulin-like growth factor type 1; GH: growth hormone.

In true or masked RAH, the medication should be progressively adjusted^16^ with the introduction of nocturnal doses of antihypertensive drugs.^18^ Patients with controlled BP on ABPM should have their therapy maintained, regardless of the office BP levels. In white-coat RAH, confirmatory ABPM needs to be performed after 3 months, and repeated every six months (if wakefulness SBP ≥ 115 mm Hg) or annually (if wakefulness SBP < 115 mm Hg).^19^

When ABPM is not available, HBPM is a good complementary method. Although it does not assess the nocturnal period and overestimates BP levels, HBPM reaches moderate agreement on the diagnosis,^20^ with high specificity and low sensitivity (Chart 2).^17^

**Chart 2:** ACC/AHA recommendations for renal artery stenosis search during coronary angiography

Clinical characteristics	Level of evidence
Beginning of hypertension < 30 years	B
Beginning of severe hypertension > 55 years	B
Accelerated/malignant hypertension	C
Resistant hypertension	C
Uremia or renal function worsening after use of ACEI or ARB (> 30% drop in glomerular filtration)	B
Atrophic kidney of unknown cause or size discrepancy between the two kidneys > 1.5 cm	B
Unexpected sudden pulmonary edema (mainly in uremic patients)	B

## Treatment

### Non-pharmacological treatment

The NPT is aimed at:

Encouraging lifestyle changes: reduction in salt intake (up to 2.0 g of sodium/day); DASH diet; body weight loss (BMI < 25 kg/m^2^); physical activity; smoking cessation; and moderate alcohol intake;^1,3,21,22^

Suspending substances that raise BP.^1,3^

### Pharmacological treatment

The basic principle of the pharmacological treatment is the association of antihypertensive drugs that block most pathophysiological mechanisms of BP elevation. Ideally, the following should be prescribed at full-tolerated dose and at proper intervals: a DIU, a RAAS inhibitor, and a dihydropyridine CCB. In certain situations, such as CAD, CHF and tachyarrhythmias, a BB can replace a CCB in the initial therapeutic regimen with 3 medications.

The correct use of DIUs to ensure control of volemic expansion is essential, and more than half of the patients can meet the BP target with DIU optimization.^13^ Chlorthalidone is superior to hydrochlorothiazide.^23^ For stage 4 or 5 CKD patients, loop DIUs should be used and administered at least twice a day. Spironolactone, an aldosterone antagonist, is the choice for the fourth drug in patients with true RAH, enabling a mean reduction of 15-20 mm Hg in SBP, and of 7-10 mm Hg in DBP, at doses of 25-50 mg/day.^24^ However, up to 20-30% of the patients might not tolerate its use, because of renal function worsening, hyperpotassemia, gynecomastia or mastalgia. In such cases, amiloride can be used (5-10 mg/day), but with an apparently lower BP response.^25^ The use of clonidine as the fourth drug is being assessed in the Brazilian ReHOT study, considering the sympathetic and RAAS activity measurements as possible predictors of the best therapeutic response to clonidine and spironolactone, respectively.^4^

In patients not reaching BP control on ABPM after the addition of spironolactone, BBs (mainly those with vasodilating effect) are the fifth drugs, if not contraindicated. Central alpha-agonists (clonidine and alpha methyldopa), direct vasodilators (hydralazine and minoxidil), or central agonists of imidazoline receptors are usually used as the sixth and seventh drugs. In addition, associations of multiple DIUs (thiazide DIUs, loop DIUs and spironolactone), especially in the presence of edema, or dihydropyridine and non-dihydropyridine CCBs can be used in the most critically ill patients.

Chronotherapy guided by ABPM, with the nocturnal administration of at least one antihypertensive drug, could improve BP control and reverse the unfavorable non-dipping pattern in those patients, in addition to reducing CV morbidity and mortality (Chart 3).^18^

**Chart 3:** Clinical indicators of probable renovascular hypertension

Probability	Clinical characteristics
Low (0.2%)	Uncomplicated borderline or mild/moderate AH
Intermediate (5-15%)	Severe or resistant AH Recent AH < 30 years or > 50 years Presence of abdominal murmur Asymmetry of radial or carotid pulses Moderate AH associated with smoking or atherosclerosis in another site (coronary or carotid) Undefined renal functional deficit Exaggerated BP response to ACEIs
High (25%)	Severe or resistant AH with progressive renal failure Accelerated or malignant AH Sudden APE ACEI-induced creatinine increase Asymmetry of renal size or function

## New therapeutic strategies

New strategies are being developed, but are still experimental. Although safe, they are not better than the conventional treatment, and should only be used in truly resistant patients (Chart 4).

**Chart 4:** Medicines and illicit and licit drugs related to AH development or worsening

Drug class	Effect on BP and frequency	Suggested action
**Immunosuppressants** Cyclosporine, tacrolimus	Intense and frequent	ACEI and CCB (nifedipine/amlodipine). Adjust serum level. Reassess options
**Anti-inflammatory agents** Glucocorticoid	Variable and frequent	
Non-steroids (1 and 2 cyclo-oxygenase inhibitors)	Occasional, very relevant with continuous use	Salt restriction, DIUs, decrease dose Observe renal function, use for a short period
**Anorexigenic/satiety drugs** Diethylpropion and others	Intense and frequent	Suspension or dose reduction
Sibutramine	Intermediate, little relevance	Assess BP reduction with weight loss
Vasoconstrictors, including ergot derivatives	Variable, transient	Use for a determined short period
**Hormones** Human erythropoietin	Variable and frequent	Assess hematocrit and dose weekly
Oral contraceptives	Variable, prevalence of up to 5%	Assess method replacement with an expert
Estrogen-replacement therapy (conjugated estrogens and estradiol)	Variable	Assess risk and cost-benefit
GH (adults)	Variable, dose-dependent	Suspension
**Antidepressant drugs** Monoamine-oxidase inhibitors	Intense, infrequent	Approach as adrenergic crisis
Tricyclics	Variable and frequent	Approach as adrenergic crisis
**Illicit drugs and alcohol** Amphetamine, cocaine and derivatives	Acute, intense effect Dose-dependent	Approach as adrenergic crisis
Alcohol	Variable and dose-dependent Very prevalent	See non-pharmacological treatment

### Direct and chronic stimulation of carotid sinus baroreceptors

The Rheos system is a programable device, like a pacemaker, surgically implanted, consisting in a generator of impulses that activate the carotid baroreceptors via radiofrequency. The Rheos Pivotal Trial has not detected significant long-term benefits.^26^

### Renal sympathetic denervation

Percutaneous transluminal renal sympathetic denervation through a catheter has been mainly assessed in the SYMPLICITY studies conducted in RAH patients. Recent meta-analyses^27,28^ have not confirmed the initially promising results.

### Use of CPAP

The antihypertensive effect of CPAP is controversial. However, as an auxiliary treatment in patients with OSAHS, mainly those who tolerate its use for more than 4 hours/night, there is evidence that it can help to reestablish the dipping pattern.^29^

### Central iliac arteriovenous anastomosis

The ROX Control HTN study^30^ has shown promising results with significant reductions in BP levels and in hypertensive complications of patients with central iliac arteriovenous anastomosis with the coupler device.

## Prognosis

A retrospective cohort study performed from a North American registry indicates that, after beginning the antihypertensive treatment, the apparent RAH incidence (uncontrolled BP with 3 medications) is 0.7/100/patients-year, and those patients' relative risk for CV events is 1.47 (95% confidence interval: 1.33-1.62).^31^ A prospective study with 556 resistant hypertensives (follow-up of 4.8 years) has shown that uncontrolled ABPM and lack of nocturnal dipping are important markers of CV risk.^32^ The apparent RAH condition is considered of independent risk for the occurrence of CV events. (GR: IIa; LE: C). Performing ABPM is recommended to establish the prognosis of hypertensives with true RAH. (GR: IIa; LE: C).

## References

[1] Calhoun DA, Jones D, Textor S, Goff DC, Murphy TP, Toto RD (2008). Resistant hypertension: diagnosis, evaluation, and treatment: a
scientific statement from the American Heart Association Professional
Education Committee of the Council for High Blood Pressure
Research. Hypertension.

[2] Judd E, Calhoun DA (2014). Apparent and true resistant hypertension: definition, prevalence
and outcomes. J Hum Hypertens.

[3] Alessi A, Brandão AA, Coca A, Cordeiro AC, Nogueira AR, Diógenes de Magalhães F (2012). First Brazilian position on resistant
hypertension. Arq Bras Cardiol.

[4] Krieger EM, Drager LF, Giorgi DM, Krieger JE, Pereira AC, Barreto-Filho JA, ReHOT Investigators (2014). Resistant hypertension optimal treatment trial: a randomized
controlled trial. Clin Cardiol.

[5] Calhoun DA, Booth 3rd JN, Oparil S, Irvin MR, Shimbo D, Lackland DT (2014). Refractory hypertension: determination of prevalence, risk
factors, and comorbidities in a large, population-based
cohort. Hypertension.

[6] Pedrosa RP, Drager LF, Gonzaga CC, Sousa MG, de Paula LK, Amaro AC (2011). Obstructive sleep apnea: the most common secondary cause of
hypertension associated with resistant hypertension. Hypertension.

[7] de Faria AP, Modolo R, Fontana V, Moreno H (2014). Adipokines; novel players in resistant
hypertension. J Clin Hypertens (Greenwich).

[8] Figueiredo VN, Yugar-Toledo JC, Martins LC, Martins LB, de Faria AP, de Haro Moraes C (2012). Vascular stiffness and endothelial dysfunction: correlations at
different levels of blood pressure. Blood Press.

[9] de la Sierra A, Segura J, Banegas JR, Gorostidi M, de la Cruz JJ, Armario P (2011). Clinical features of 8295 patients with resistant hypertension
classified on the basis of ambulatory blood pressure
monitoring. Hypertension.

[10] Egan BM, Zhao Y, Li J, Brzezinski WA, Todoran TM, Brook RD (2013). Prevalence of optimal treatment regimens in patients with
apparent treatment-resistant hypertension based on office blood pressure in
a community-based practice network. Hypertension.

[11] Burnier M, Wuerzner G, Struijker-Boudier H, Urquhart J (2013). Measuring, analyzing, and managing drug adherence in resistant
hypertension. Hypertension.

[12] Muxfeldt ES, de Souza F, Salles GF (2013). Resistant hypertension: a practical clinical
approach. J Hum Hypertens.

[13] Garg JP, Elliott WJ, Folker A, Izhar M, Black HR, RUSH University Hypertension Service (2005). Resistant hypertension revisited: a comparison of two university
based cohorts. Am J Hypertens.

[14] Muxfeldt ES, Margallo VS, Guimarães GM, Salles GF (2014). Prevalence and associated factors of obstructive sleep apnea in
patients with resistant hypertension. Am J Hypertens.

[15] Calhoun DA (2013). Hyperaldosteronism as a common cause of resistant
hypertension. Annu Rev Med.

[16] Muxfeldt ES, Salles GF (2013). How to use ambulatory blood pressure monitoring in resistant
hypertension. Hypertens Res.

[17] Muxfeldt ES, Barros GS, Viegas BB, Carlos FO, Salles GF (2015). Is home blood pressure monitoring useful in the management of
patients with resistant hypertension. Am J Hypertens.

[18] Hermida RC, Ayala DE, Fernández JR, Calvo C (2008). Chronotherapy improves blood pressure control and reverts the
nondipper pattern in patients with resistant hypertension. Hypertension.

[19] Muxfeldt ES, Fiszman R, de Souza F, Viegas B, Oliveira FC, Salles GF (2012). Appropriate time interval to repeat ambulatory blood pressure
monitoring in patients with white-coat resistant
hypertension. Hypertension.

[20] Nasothimiou EG, Tzamouranis D, Roussias LG, Stergiou GS (2012). Home versus ambulatory blood pressure monitoring in the diagnosis
of clinic resistant and true resistant hypertension. J Hum Hypertens.

[21] Pimenta E, Gaddam KK, Oparil S, Aban I, Husain S, Dell'Italia LJ (2009). Effects of dietary sodium reduction on blood pressure in subjects
with resistant hypertension: results from a randomized trial. Hypertension.

[22] Guimaraes GV, de Barros Cruz LG, Fernandes-Silva MM, Dorea EL, Bocchi EA (2014). Heated water-based exercise training reduces 24-hour ambulatory
blood pressure levels in resistant hypertensive patients: a randomized
controlled trial (HEx trial). Int J Cardiol.

[23] Ernst ME, Carter BL, Zheng S, Grimm Jr RH (2010). Meta-analysis of dose-response characteristics of
hydrochlorothiazide and chlorthalidone: effects on systolic blood pressure
and potassium. Am J Hypertens.

[24] Liu G, Zheng XX, Xu YL, Lu J, Hui RT, Huang XH (2015). Effect of aldosterone antagonists on blood pressure in patients
with resistant hypertension: a meta-analysis. J Hum Hypertens.

[25] Lane DA, Beevers DG (2007). Amiloride 10 mg is less effective than spironolactone 25 mg in
patients with hypertension resistant to a multidrug regime including an
angiotensin-blocking agent. J Hypertens.

[26] Bakris GL, Nadim MK, Haller H, Lovett EG, Schafer JE, Bisognano JD (2012). Baroreflex activation therapy provides durable benefit in
patients with resistant hypertension: results of long-term follow-up in the
Rheos Pivotal Trial. J Am Soc Hypertens.

[27] Fadl Elmula F, Jin Y, Larstorp AC, Persu A, Kjeldsen SE, Staessen JA (2015). Meta-analysis of five prospective and randomized trials of renal
sympathetic denervation on office and ambulatory systolic blood pressure in
treatment resistant hypertension. J Hypertens.

[28] Fadl Elmula FE, Jin Y, Yang WY, Thijs L, Lu YC, Larstorp AC, European Network Coordinating Research On Renal Denervation
Consortium (2015). Meta-analysis of randomized controlled trials on renal
denervation in treatment -resistant hypertension. Blood Press.

[29] Martinez-Garcia MA, Capote F, Campos-Rodriguez F, Lloberes P, Díaz de Atauri MJ, Somoza M, Spanish Sleep Network (2013). Effect of CPAP on blood pressure in patients with obstructive
sleep apnea and resistant hypertension: the HIPARCO randomized clinical
trial. JAMA.

[30] Lobo MD, Sobotka PA, Stanton A, Cockcroft JR, Sulke N, Dolan E, ROX CONTROL HTN Investigators (2015). Central arteriouvenous anastomosis for the treatment of patients
with uncontrolled hypertension (the ROX CONTROL HTN Study): a randomised
controlled trial. Lancet.

[31] Daugherty SL, Powers JD, Magid DJ, Tavel HM, Masoudi FA, Margolis KL (2012). Incidence and prognosis of resistant hypertension in hypertensive
patients. Circulation.

[32] de Souza F, Muxfeldt ES, Salles GF (2012). Prognostic factors in resistant hypertension: implications for
cardiovascular risk stratification and therapeutic
management. Expert Rev Cardiovasc Ther.

